# VASO (Vitamin D and Arthroplasty Surgery Outcomes) study - supplementation of vitamin D deficiency to improve outcomes after total hip or knee replacement: study protocol for a randomised controlled feasibility trial

**DOI:** 10.1186/s13063-017-2255-2

**Published:** 2017-11-02

**Authors:** Rory J. M. Morrison, Deborah Bunn, William K. Gray, Paul N. Baker, Craig White, Amar Rangan, Kenneth S. Rankin, Mike R. Reed

**Affiliations:** 1 Department of Orthopaedics, Northumbria Healthcare NHS Foundation Trust, Woodhorn Lane, Ashington, Northumberland NE63 9JJ UK; 2 University of Newcastle, Newcastle upon Tyne, NE1 7RU UK; 3 Research and Development, Northumbria Healthcare NHS Foundation Trust, Rake Lane, North Shields, NE29 8NH UK; 4 Department of Orthopaedics, South Tees Hospitals NHS Foundation Trust, Marton Lane, Middlesbrough, TS4 3BW UK; 5 Department of Health Sciences, University of York, Heslington, York, YO10 5DD UK; 6 Faculty of Medical Sciences & NDORMS, University of Oxford, Oxford, OX3 7LD UK

**Keywords:** Vitamin D, Cholecalciferol, Deficiency, PROMs, Arthroplasty, THR, TKR, Hip, Knee, Replacement

## Abstract

**Background:**

Vitamin D deficiency has been linked to poor outcomes after total hip replacement (THR) or total knee replacement (TKR), including lower patient-reported outcome measures (PROMs), peri-prosthetic infection and longer hospital stay. We present a randomised feasibility trial protocol designed to prospectively investigate the influence of vitamin D testing, and subsequent supplementation for deficiency, prior to THR/TKR.

**Methods/design:**

One hundred adult patients undergoing primary THR/TKR for osteoarthritis at two NHS hospital trusts in North East England will be recruited. Exclusion criteria include lack of mental capacity, revision surgery, participants already taking vitamin D/calcium supplements, or a known contraindication to vitamin D treatment. Participants will be ineligible for the trial if they have an estimated glomerular filtration rate < 30 ml/minute. We will measure patients’ vitamin D levels at baseline, and those identified as deficient (vitamin D < 50 nmol/L) will be randomised to receive either vitamin D supplementation or no supplementation prior to, and for 6 months following, surgery. Patients with a normal vitamin D level (≥50 nmol/L) will receive no supplementation. Vitamin D levels will be rechecked on the day of surgery and again at 6 months. Patients will also complete a lifestyle questionnaire, as well as the Oxford hip or knee and EQ-5D-3 L PROM questionnaires, at baseline and at 6 months following surgery. The aims are to determine the feasibility of the methodology and to gather data to inform the conduct of a future, larger trial to investigate if supplementation with vitamin D, in those who are deficient, prior to THR/TKR improves outcomes as measured by PROM scores.

**Discussion:**

Previous reports have measured vitamin D levels and correlated this to outcome, but we can find no randomised trial in which researchers investigated the effect of supplementation. The aim of this trial is to determine if vitamin D deficiency is a modifiable risk factor for poor outcome after THR/TKR.

**Trial registration:**

ISRCTN Registry, ISRCTN14533082. Registered on 3 April 2017.

**Electronic supplementary material:**

The online version of this article (doi:10.1186/s13063-017-2255-2) contains supplementary material, which is available to authorized users.

## Background

The extra-skeletal effects of vitamin D are well documented, affecting almost every cell type through the vitamin D receptor, and deficiency has been linked to a myriad of conditions, including cancer, auto-immune disease, cardio-respiratory disease and depression [1]. The vitamin is synthesised in the skin in response to sunlight; however, for at least 6 months of the year, the United Kingdom is located above the latitude where this can be achieved [2]. Consequently, insufficient levels during the winter and spring months are reported in > 50% of adults over 65 years of age [3].

Vitamin D deficiency has been linked to poorer patient-reported outcome measures (PROMs) following total hip replacement (THR) and total knee replacement (TKR). In 2010, researchers in a study comprising 62 patients reported that those with vitamin D deficiency (25-hydroxyvitamin D [25-OH vitamin D] < 40 nmol/L) had lower Harris Hip Scores before and after surgery than those with a sufficient level (25-OH vitamin D > 40 nmol/L) [4]. A similar result was found in patients undergoing TKR [5]. Shin et al. reported a statistically significant lower Knee Society score, as well as a longer 6-m walk time, following TKR surgery in patients with vitamin D deficiency than in those with a normal level [6].

Lee et al. [7] suggested a link between vitamin D deficiency and post-operative pain. The authors found that, at 3 months following TKR surgery, 13.8% of those patients with pre-operative deficiency were more likely to report ongoing moderate to severe pain than those with sufficient levels (5.9%; *p* = 0.05), and the role of vitamin D in the modulation of anti-inflammatory cytokines was suggested as a possible reason for this.

In a German study of over 1000 arthroplasty patients, vitamin D deficiency was associated with a significantly longer stay following surgery, by 4.3 days (mean 15.6 vs. 11.3 days), even when adjusting for confounders in multivariable analysis [8]. The same authors reported a significantly greater prevalence of vitamin D deficiency in patients presenting at their unit for revision surgery due to peri-prosthetic joint infection, compared with those presenting with aseptic loosening or for primary surgery [9]. Their conclusion, like that of others, is recommendation of a randomised trial to investigate the effect of vitamin D supplementation prior to THR/TKR in a bid to improve outcomes. To date, to our knowledge, no such trial has been performed or registered.

On the basis of National Joint Registry data, 190,000 primary THR/TKR procedures were carried out in England, Wales and Northern Ireland in 2016 [10]. Some patients remain unhappy following joint replacement surgery, with up to 20% of TKR patients reporting dissatisfaction [11, 12], and the origin of a negative outcome may be multi-factorial. PROM scores are measured in the NHS prior to and at 6 months following surgery to assess outcomes following THR/TKR, with financial implications for NHS trusts if they are a negative outlier, defined as 3 SD below the mean PROM improvement. NHS PROMs are currently assessed using the Oxford hip or knee score, as well as the EQ-5D-3 L, a generic measure of health status.

## Methods/design

### Aim

The aim of this prospective, randomised controlled trial is to determine the feasibility of the methodology and to gather data to inform the conduct of a future, larger trial to investigate if supplementation with vitamin D, in those who are deficient, prior to THR/TKR improves outcomes as measured by PROM scores.

### Ethics, consent and permissions

Northumbria Healthcare NHS Foundation Trust is the trial sponsor. The trial will be conducted in accordance with the Declaration of Helsinki. The trial has received a favourable ethical opinion from the Yorkshire and the Humber – Bradford Leeds Research Ethics Committee (17/YH/0067) and has Health Research Authority approval (Integrated Research Application Service 216934). It has National Institute for Health Research portfolio status (Central Portfolio Management System identifier 33969) and was prospectively registered on 3 April 2017 with the International Standard Registered Clinical/soCial sTudy Number (ISRCTN) database (ISRCTN14533082). The current protocol is version 1, dated 31 January 2017, and any changes to the protocol will be communicated to all relevant parties, including patients. We have received charitable funding from Orthopaedic Research UK to support this study.

### Participant recruitment

**Fig. 1:**
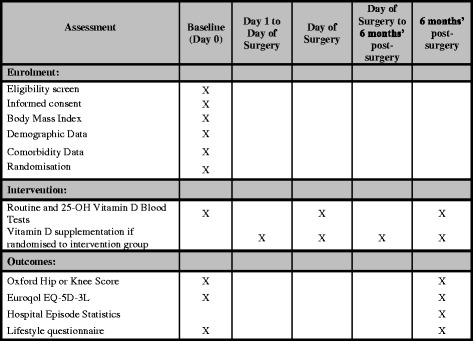
Standard Protocol Items: Recommendations for Interventional Trials (SPIRIT) checklist. *25-OH Vitamin D* 25-Hydroxyvitamin D

In this feasibility trial, we will recruit a total of 100 participants from 2 NHS hospital trusts in North East England: Northumbria Healthcare NHS Foundation Trust and South Tees Hospitals NHS Foundation Trust. Participant recruitment was expected to commence by May 2017, and the trial is due to last 20 months. This assumes 2 months for patient recruitment, up to 3 months between recruitment and surgery, 6 months to complete post-operative PROM scores, 6 months for data retrieval and analysis, and 3 months to write the study report. Figure 1 displays the trial schedule, and a Standard Protocol Items: Recommendations for Interventional Trials (SPIRIT) checklist is included as Additional file 1.

### Eligibility

Participants will be older than 18 years of age and be scheduled for primary THR/TKR performed for a diagnosis of osteoarthritis. Exclusion criteria include participants who lack the mental capacity to comply with study procedures, participants undergoing revision surgery, participants already taking vitamin D supplements, participants with a known contraindication to vitamin D treatment (such as a previous diagnosis of sarcoidosis or hyperparathyroidism), and participants with an allergy to vitamin D. Participants will be ineligible for the trial if they have renal impairment with an estimated glomerular filtration rate (eGFR) < 30 ml/minute.

### Informed consent

Eligible participants will be identified in the outpatient clinic of their treating surgeon once they have been added to the waiting list for surgery. The research team will inform potential participants about the trial and provide them with a patient information sheet. Willing participants will be screened for eligibility by good clinical practice (GCP)-trained research staff, and have the opportunity to ask any questions they may have about the trial, before giving written informed consent for participation (Additional file 2). Patients will be given up to 1 week to make a decision regarding their involvement in the trial; if they are happy to give consent straight away, then this will be allowed. Patients will be free to withdraw from the study at any time without having to give a reason. They will not be eligible to participate in other clinical trials during the study period.

### Baseline assessment

Participants will have routine baseline blood tests performed, including full blood count, urea and electrolytes, eGFR, liver function tests, calcium and albumin, as well as having their serum 25-OH vitamin D level measured. These tests will be analysed pragmatically using the routine laboratory methods available at each NHS Trust hospital. Northumbria use the cobas e 601 Total 25-OH vitamin D immunochemiluminescence assay (Roche Diagnostics International Ltd., Rotkreuz, Switzerland), which has a detection range of 7.5–175 nmol/L and a laboratory-quoted coefficient of variation of 7.7%. At South Tees, the IDS-iSYS 25-Hydroxy Vitamin D^s^ immunoassay (Immuno Diagnostic Systems [IDS], Boldon, UK) is used, which has a detection range of 18–313 nmol/L and a quoted coefficient of variation up to 11.6%.

Patients will also be asked if they give their consent for an extra sample of blood to be taken and stored anonymously at Newcastle University for research purposes only, including in future research trials. Participants will provide basic demographic data and complete a questionnaire regarding diet and lifestyle to assess their exposure to environmental and dietary vitamin D. Whilst we could not find a validated questionnaire to assess diet and lifestyle influences, the questions we will ask are similar to those reported by Lee et al. [13]. Answers will be recorded in the case report form (CRF), and patients will be allocated a trial identifier that does not contain any patient-identifiable data.

As part of their routine care, participants complete baseline PROMs, which are either the Oxford hip or knee score, and the EQ-5D-3 L questionnaires (Appendix 1). These are routine measures collected by the NHS to assess the quality of care experienced by patients undergoing THR/TKR. These questionnaires, which are used to gather routinely collected data, will be obtained for this trial.

### Randomisation

Those participants with vitamin D deficiency (< 50 nmol/L) will be randomised to receive either vitamin D supplementation or no supplementation. Research nurses recruiting participants into the trial will call the research and development (R&D) department at the sponsor site by telephone to obtain the participant’s randomised group allocation. The central R&D administrator will provide this information from a randomisation log generated by the website www.randomization.com. Randomisation will be stratified by study site, and patients will be allocated to either vitamin D supplementation or no supplementation using a 1:1 ratio at both sites. The R&D administrator will provide written confirmation of group allocation using secure nhs.net email.

### Intervention group

Those patients who are randomised to receive treatment will receive oral cholecalciferol according to their vitamin D level. Patients with insufficiency (25–49 nmol/L) will receive 1600 IU/day oral cholecalciferol until 6 months following surgery. Patients with deficiency (< 25 nmol/L) will receive 20,000 IU of oral cholecalciferol twice per week for 8 weeks, then receive 1600 IU/day oral cholecalciferol until 6 months following surgery. Patients’ calcium levels will be re-checked to monitor for hypercalcaemia, and vitamin D treatment will be stopped if this is noted. Patients will not be blinded to their treatment allocation.

### Control groups

Those participants with a normal vitamin D level (≥ 50 nmol/L) will be eligible for inclusion in the trial, but not for randomisation. Along with those patients with vitamin D deficiency who are randomised to receive no supplementation, they will act as control subjects. The control patients will be blinded to their vitamin D status for the duration of the trial and asked not to start taking any supplements containing vitamin D for the duration of their participation in the trial. There are therefore three groups in this trial; patients with vitamin D deficiency who do not receive supplementation, patients with vitamin D deficiency who receive supplementation, and patients with sufficient vitamin D levels. Not providing vitamin D supplementation is current standard practice in the participating NHS trusts, so the control group will, in effect, receive standard care.

### Surgical procedure

All participants will have blood tests, including re-measurement of 25-OH vitamin D on the day of surgery, prior to the start of the operation. Patients receiving vitamin D supplementation will also have their calcium level re-checked to monitor for hypercalcaemia. They will undergo primary THR/TKR under the care of their consultant orthopaedic surgeon, and no specific guidance is given with regard to choice of implant, surgical approach, closure, peri-operative care, rehabilitation or clinical follow-up; the approach is pragmatic and should be routine care for that unit.

### Post-operative follow-up

For the purposes of the trial, all participants will be seen again at 6 months following surgery to repeat the diet and lifestyle questionnaire and to re-measure their 25-OH vitamin D level. They will also be reminded about completion of the routine 6-month post-operative PROM scores sent by the NHS. Those participants who were randomised to receive treatment will also be asked to complete an adherence to supplementation questionnaire and return their vitamin D supplement packets.

### Outcomes

The overall aim of this study is to determine if it is feasible to run a large, multi-centre clinical trial to investigate the effect of vitamin D supplementation on outcome following THR/TKR. We will analyse the trial processes, which are key to the future study. We will record the number of eligible patients per site as well as recruitment, retention and refusal rates. The proportion of patients undergoing primary THR/TKR who did not meet the eligibility criteria will also be calculated to ensure that this is not too limiting.

The primary clinical outcome measurement for the trial is the effect of vitamin D on health gain following THR/TKR. Health gain is calculated as the difference between pre-operative and post-operative PROM scores. Condition-specific health gain is measured using either the Oxford hip or knee scores, whilst generic health is measured using the EQ-5D-3 L. We will obtain the routine patient-level PROM data that is supplied to NHS trusts to determine these outcomes.

Secondary outcomes include length of hospital stay, readmission rate and complications. To determine these outcomes, we will interrogate data routinely held in patient records and hospital databases at the study sites. These databases will allow the research team to collect demographic data, co-morbidities, hospital episode statistical data (including length of stay, readmission within 30 days of surgery, return to theatre within 30 days of surgery, requirement for critical care during admission), and complication data to include 30-day medical complications, 60-day thromboembolic complications, 90-day mortality and any superficial or deep infections within the study period. Any expected or unexpected adverse events will be recorded on a trial adverse events form and reported to the sponsor. Any serious adverse events, including death, those prolonging or requiring hospitalisation, causing permanent or significant disability/incapacity, or a life-threatening condition, will be reported to the sponsor and the research ethics committee. The relationship to the study will also be determined. The sponsor has usual NHS indemnity.

### Statistical analysis

A secure Excel database (Microsoft Inc., Redmond, WA, USA) will be used to record data. Statistical analysis will be performed using IBM SPSS software (IBM, Armonk, NY, USA). We will compare health gain between groups at baseline and at 6 months, as well as mean pre-operative and post-operative PROM scores between groups. Significance will be denoted at *p* < 0.05, and we will report 95% CIs as appropriate. The statistical test chosen to compare groups will depend on the data distribution and the level of the data (nominal, ordinal or interval/ratio). For parametric data comparing two groups, Student’s *t* test for independent samples will be used. The Welch correction will be used if variances are unequal. If the distributions are not normal or the data ordinal, the Mann-Whitney *U* test will be used. The chi-square test or Fisher’s exact test, depending on observed frequencies, will be used for non-parametric data. Paired tests will be used to compare data within participants pre- and post-surgery as appropriate, with tests (e.g., paired *t* test, Wilcoxon signed-rank test) selected depending on the level and distribution of the data as described above. We recognise that outcomes are likely to be confounded with baseline characteristics (e.g., low physical activity levels, age), type of surgery (TKR/THR) and hospital site, and we will investigate the influence of these baseline confounders on outcomes using multivariable modelling (e.g., logistic regression, analysis of covariance). However, given that this is a feasibility study, and it is therefore not fully powered, such multivariable techniques will be used sparingly and only where justified (e.g., sufficient variable-to-participant ratio). The main aim of inferential analysis of clinical outcomes will be to inform a future fully powered study, rather than to draw direct inferences regarding outcomes.

Using the observed mean and SD of the PROM scores collected from this preliminary data, as well as the reported minimal clinically important difference for the Oxford hip and knee scores and EQ-5D-3 L, a sample size calculation can be performed to inform a larger, future trial. The retention rate derived from these feasibility data will be incorporated into the final sample size to allow for possible attrition. The recruitment rate and resource allocation can also be applied to the final sample size required to plan the future trial requirements. The two NHS trusts involved in this trial carried out almost 4500 hip or knee replacements in 2016. We have chosen to recruit 100 patients for this feasibility trial on the basis of an expected recruitment rate at the two NHS trusts over 2 months.

### Data management

Each site will hold data according to the Data Protection Act 1998, and data will be collated in CRFs identified by a unique identification number (i.e., the trial number) only. A trial enrolment log at the sites will list the identification numbers. All data recorded electronically will be held on password-protected NHS trust information technology systems with permission for access as detailed in the delegation log. All study files will be stored in accordance with GCP guidelines. Trial data will be sent to the sponsor via secure nhs.net email and accessed only by the study team. Study documents held by the sponsor will be retained in a secure, locked location for the duration of the trial. All essential documents, including source documents, will be retained for a minimum period of 5 years after study completion. All work will be conducted following NHS trust data protection policy.

### Trial management

The trial management group is the executive decision-making body and is responsible for the day-to-day running and management of the feasibility study. Led by the chief investigator, it will consist of the lead investigator, research nurses, and statistician. The team will meet on a monthly basis by teleconference and face-to-face at least once per year. It will monitor adherence to the trial protocols at the trial sites. Quality assurance checks will be undertaken to ensure the integrity of randomisation, study entry procedures and data collection. Safety and efficacy will be assessed using adverse event data collection. This will be analysed 6 months from the study opening to recruitment and again at 12 months.

### Participant flow

**Fig. 2:**
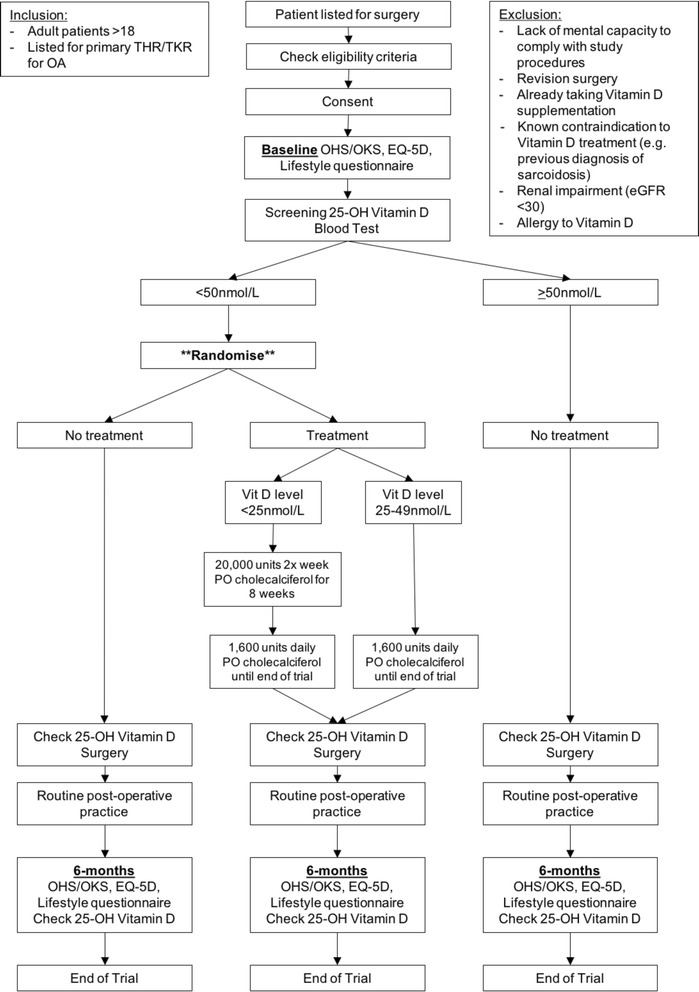
Participant flow in the Vitamin D and Arthroplasty Surgery Outcomes (VASO) study. *eGFR* estimated glomerular filtration rate, *OA* osteoarthritis, *OHS* Oxford hip score, *OKS* Oxford knee score, *PO* Per os / "oral", *THR* Total hip replacement, *TKR* Total knee replacement

The flow of participants in the trial is displayed in Fig. 2.

## Discussion

A number of studies have suggested a link between vitamin D deficiency and poor outcomes after joint replacement surgery [4–9, 14–16]. However, these studies have only measured vitamin D levels and have not prospectively observed the effect of supplementation. We can find no randomised interventional trial in the published literature to investigate if vitamin D deficiency is a modifiable risk factor and whether supplementation prior to surgery improves outcomes; this trial would therefore be the first.

For this feasibility trial, we will not delay the date of surgery until vitamin D levels have normalised. One of the questions to answer in this feasibility study is if surgery should be delayed until vitamin D levels are normalised in a definitive trial. Currently we have no good evidence that surgery should be delayed.

The design of this trial has been discussed with the Total Hip User Group, a local expert patient group consisting of patients who have undergone, despite their name, both THR and TKR. They have also had input with regard to the wording of the patient information sheet and informed consent form.

There are a number of limitations to the present feasibility study. Firstly, there are two different methods for measurement of vitamin D used between the two centres, although this is pragmatic, and patients will get repeated measurements at the same hospital with the same analyser. The trial is being conducted in only one region, and therefore geographical influences on vitamin D levels will not be accounted for. The trial period is only recruiting over a short time period, rather than 1 whole year, so seasonal variations in vitamin D level cannot be accounted for. We are not measuring other markers of bone status such as parathyroid hormone, and we are not measuring vitamin D binding protein. We are also giving those patients randomised to treatment standard doses based on their category of insufficiency, rather than treating them individually to a target. Finally, there is no placebo used for the control groups.

The results of this trial will be presented at scientific meetings and submitted for publication in relevant peer-reviewed journals. Results will also be disseminated to patients and staff involved in the trial. Published results will not contain any patient-identifiable data and will be presented for the whole group rather than for individual participants. The results of this trial will be used to inform the design of a definitive, large-scale, multi-centre randomised controlled trial.

### Trial status

The current protocol is version 1, dated 31 January 2017. Recruitment began on 4 May 2017, and the trial was still open at the time of this protocol submission. The trial is expected to run until December 2018.

### Additional files

Additional file 1: SPIRIT 2013 checklist: recommended items to address in a clinical trial protocol and related documents. (DOC 122 kb)

Additional file 2: Consent form for the VASO trial. (TIFF 489 kb)

## Appendix 1

### Patient-reported outcome measures used in the trial

1. *Oxford hip or knee score:* The Oxford hip [17] and knee [18] scores are validated 12-item questionnaires used to assess function and pain with 5 response options per question. The overall score is from 0 to 48, with the highest score indicating the best outcome.
2. *EQ-5D-3 L:* The EQ-5D-3 L [19] assesses five domains of health—mobility, self-care, usual activities, pain/discomfort and anxiety/depression. There are three levels of responses for each: no problems, some problems and extreme problems/unable to carry out. Patients are also asked to complete a visual analogue scale ranging from 0 (worst) to 100 (best) with reference to their own health state. Responses are converted to an ‘index’ score which can be compared with population norms.

## Abbreviations

25-OH Vitamin D: 25-Hydroxyvitamin D
CRF: Case report form
eGFR: Estimated glomerular filtration rate
GCP: Good clinical practice
HRA: Health Research Authority
IRAS: Integrated Research Application Service
ISRCTN: International Standard Registered Clinical/soCial sTudy Number
PROM: Patient-reported outcome measure
R&D: Research and development
SPIRIT: Standard Protocol Items: Recommendations for Interventional Trials
THR: Total hip replacement
TKR: Total knee replacement
VASO: Vitamin D and Arthroplasty Surgery Outcomes
