# Supplementary figures and images for: VASO (Vitamin D and Arthroplasty Surgery Outcomes) study - supplementation of vitamin D deficiency to improve outcomes after total hip or knee replacement: study protocol for a randomised controlled feasibility trial
Source: Trials. 2017 Nov 2;18:514. doi: 10.1186/s13063-017-2255-2 (PMC5669000; doi:10.1186/s13063-017-2255-2)

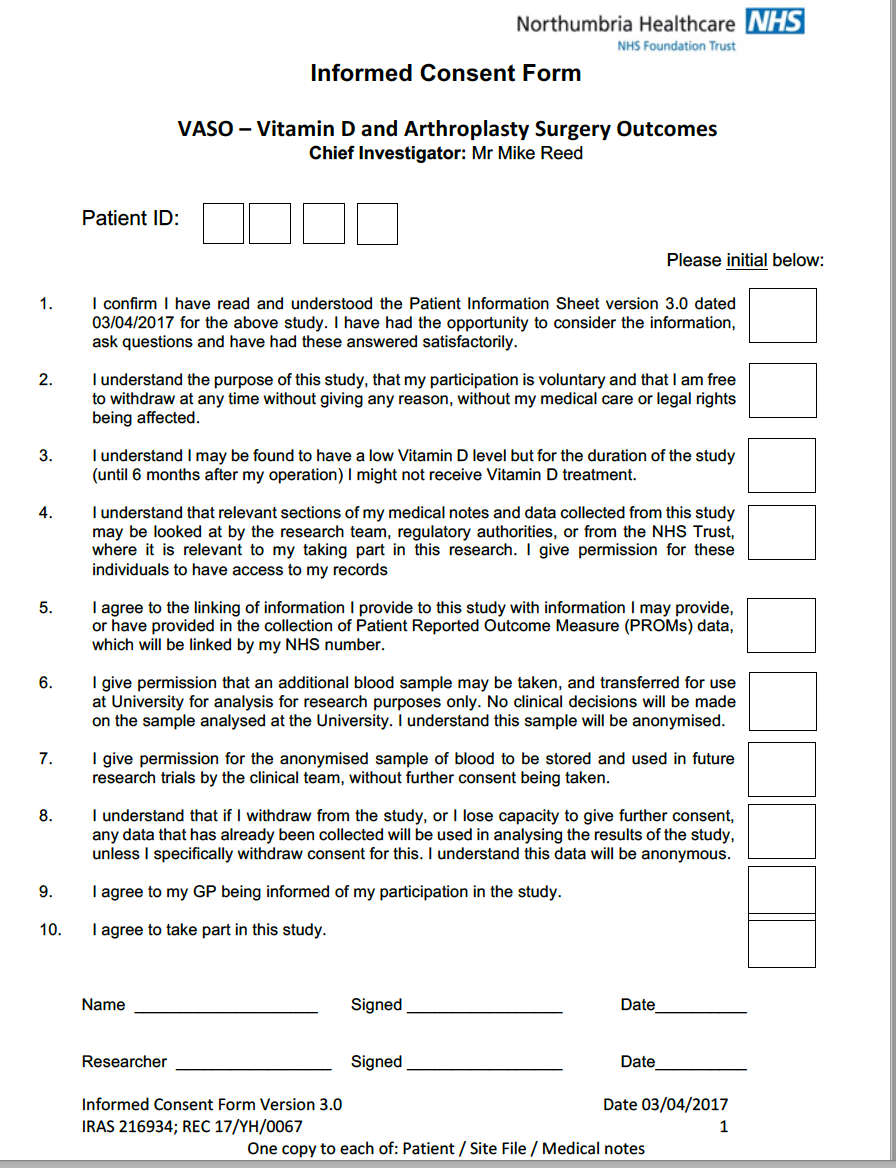

Supplement: Supplementary file 2 — Consent form for the VASO trial. (TIFF 489 kb) [file 13063_2017_2255_MOESM2_ESM.tiff]
